# Efficacy of Cognitive Behavioral Therapy on Mood Disorders, Sleep, Fatigue, and Quality of Life in Parkinson's Disease: A Systematic Review and Meta-Analysis

**DOI:** 10.3389/fpsyt.2021.793804

**Published:** 2021-12-13

**Authors:** Fangyi Luo, Mengfei Ye, Tingting Lv, Baiqi Hu, Jiaqi Chen, Junwei Yan, Anzhe Wang, Feng Chen, Ziyi He, Zhinan Ding, Jian Zhang, Chao Qian, Zheng Liu

**Affiliations:** ^1^Department of Psychiatry, Shaoxing Seventh People's Hospital, Affiliated Mental Health Center, Medical College of Shaoxing University, Shaoxing, China; ^2^Department of Behavioral Neurosciences, Science Research Center of Medical College, Shaoxing University, Shaoxing, China; ^3^Department of Neurology, Shaoxing Hospital, China Medical University, Shaoxing, China; ^4^Laboratory of Forensic Toxicology, Judicial Identification Center of Shaoxing University, Shaoxing, China

**Keywords:** cognitive behavioral therapy, Parkinson's disease, non-motor symptoms, quality of life, meta-analysis

## Abstract

**Objective:** The aim of this study was to perform a quantitative analysis to evaluate the efficacy of cognitive behavioral therapy (CBT) on mood disorders, sleep, fatigue, and its impact on quality of life (QOL) in Parkinson's Disease (PD).

**Methods:** We searched for randomized controlled trials in three electronic databases. Fourteen studies, including 507 patients with PD, met the inclusion criteria. We determined the pooled efficacy by standard mean differences and 95% confidence intervals, using *I*^2^ to reveal heterogeneity.

**Results:** The result showed CBT had a significant effect on depression [−0.93 (95%CI, −1.19 to −0.67, *P* < 0.001)] and anxiety [−0.76 (95%CI, −0.97 to −0.55, *P* < 0.001)]. Moderate effect sizes were noted with sleep disorders [−0.45 (95% CI, −0.70 to −0.20, *P* = 0.0004)]. There was no evident impact of CBT on fatigue or QOL. We found an intervention period >8 weeks was advantageous compared with <8 weeks, and CBT implemented in non-group was more effective than in group. Between the delivery methods, no significant difference was found.

**Conclusion:** We found that CBT in patients with PD was an efficacious therapy for some non-motor symptoms in PD, but not efficacious for fatigue and QOL. These results suggest that CBT results in significant improvement in PD and should be used as a conventional clinical intervention.

## Highlights

- The aim of this study was to perform a quantitative analysis to evaluate the efficacy of CBT on common non-motor symptoms and its impact on QOL in PD.
- CBT in patients with PD was an efficacious therapy for some non-motor symptoms in PD such as depression, anxiety, sleep, and so on, but not efficacious for fatigue. The improvement in quality of life was not evident when the intervention was <10 weeks.
- There was no significant difference in the efficacy of the on-line and off-line interventions. In anxiety and depression, treatment in individual had better outcomes, while the effect of group or individual was similar in the sleep.
- CBT should be considered as a standard clinical treatment for non-motor symptoms of PD to relieve the suffering patients, and more convenient and efficient forms should be explored.

## Introduction

Parkinson's Disease (PD), a common neurodegenerative disorder with motor and non-motor symptoms that is second in prevalence to Alzheimer's disease, affects more than 1% of the world's population (1). Compared with motor symptoms (resting tremor, bradykinesia, rigidity, and gait disturbances), non-motor symptoms (mood disturbances, fatigue, apathy, and sleep disorders) are often clinically underappreciated (2, 3). Of these non-motor symptoms, the prevalence of mixed depression and anxiety reaches 50% and is more commonly found in patients with PD than in the general population (4). Non-motor symptoms also have a negative effect on quality of life (QOL) for patients and their families, thus addressing these symptoms is urgent (5).

The treatment modalities for PD are expanding rapidly. Among all of the treatment options, pharmacotherapy remains first-line (6). However, the side effects of long-term medication use are serious and in the presence of symptoms, frequent physical monitoring is needed which makes pharmacotherapy restrictive (7, 8). As a widely used antidepressant drug, SSRIs is certainly effective while its side effects are not negligible, for example gastrointestinal symptoms, neurological symptoms, and sexual dysfunction which may affect daily life. Cognitive behavioral therapy (CBT) is a non-pharmacotherapy to change psychological state and improve psychological problems by changing the patients' cognition and behavior that has been shown to be effective for mental illness (9, 10). The anterior cingulate and orbital frontal cortex of brain associated with the regulation of emotion are activated by CBT (11). A previous meta-analysis evaluating the effects of CBT on anxiety in children confirmed that the safety of CBT was reliable (12). No serious adverse events related to CBT was reported in our included studies, and there was no evidence that withdrawal was due to the CBT intervention implemented in the study. Administration of regular sessions of CBT, which incorporates tailored interventions of relaxation training, thought monitoring and restructuring, sleep hygiene, worry control, and others, has resulted in symptom improvements in patients with PD (13).

Koychev and Okai (14) have reported the comprehensive benefits of CBT on the non-motor symptoms of PD in a clinical review, which assessed three psychiatric manifestations using four uncontrolled studies and two case series. Because of the barriers to receiving psychotherapy like CBT, such as lack of trained clinicians and fear of stigma, clinicians who have implemented the original form of CBT have encountered some obstacles (15, 16). Therefore, CBT was further developed and more viable forms have been adopted widely to make the implementation of CBT more convenient and efficient, for example, intervention can be performed on-line rather than in person or may be administered in a group rather than with individuals. These treatments have been shown to have different effects on non-motor symptoms of Parkinson's disease.

Currently, several previous meta-analysis studies have, respectively, evaluated the efficacy of CBT in treating depression, anxiety and insomnia (17–19), but there has been no comprehensive quantitative analysis of the efficacy of CBT on non-motor symptoms of PD. The aim of this study was to perform a quantitative analysis of existing randomized controlled trials (RCT) to estimate the efficacy of CBT on non-motor symptoms and quality of life in PD, and ascertain which forms and duration of CBT intervention are best in an effort to inform clinical treatment.

## Methods

### Search Methods

We performed a systematic search of RCTs on PubMed, Embase, and the Cochrane Library up until August 1st, 2021. The following keywords and their synonyms were used: Parkinson's Disease, cognitive behavioral therapy, mindfulness-based cognitive therapy, acceptance and commitment therapy and dialectical behavior therapy. We attempted to access full texts of the retrieved literature. The specific retrieval strategy was attached in the Appendix (Search Strategy) (Supplementary Material).

### Inclusion and Exclusion Criteria

The inclusion criteria were as follows: (1) The study designed as a RCT (Random methods can be a computer-based random number generator, sealed envelopes including numbers and others); (2) participants had PD with any kind of non-motor symptoms (including depression, anxiety, sleep disorders, apathy, and others) without limiting the severity of the symptoms; (3) the intervention was CBT and its derivative therapy (both need to include the core CBT skills and specific instructions) and (4) the outcome was evaluated by clinically approved scales.

The exclusion criteria were as follows: (1) patients with the following conditions: severe dementia or physical impairment, acute suicide intent, psychosis, drug abuse, and uncontrolled bipolar disorder; (2) studies about motor symptoms (resting tremor, bradykinesia, rigidity, gait disturbances, and others) in PD; (3) lack of raw data or absence of data; and (4) no English publications or duplicated publications.

### Study Screening and Data Extraction

Two authors (FL and TL) performed the search independently and excluded irrelevant articles according to the criteria above, and then accessed full-text articles where available. Both the authors evaluated the eligible articles, respectively, and gathered relevant information into a pre-designed data form. The form included author, year published, country of origin, study design, comorbidities, duration of PD, Hoehn and Yahr status, non-motor symptoms, mean age of patients, sample size, and intervention for the two groups, CBT completion, frequency of CBT, measurement scales used, and study duration. Another data form was used for the outcome data gleaned from all of the scales used in the selected studies. Discrepancies were resolved by consensus discussion. When study details were incongruously documented between the two evaluating authors, a third author re-evaluated the study in question.

### Outcome

The outcome of our study was assessed by clinically approved scales. These scales evaluated all non-motor symptoms that we included. Depression severity was evaluated with the Hamilton Depression Rating Scale (20), the Beck Depression Inventory (21), the Geriatric Depression Scale-15 item short (22), the Patient Health Questionnaire-9 (23), and some others. Anxiety severity was measured with the Hamilton Anxiety Rating Scale score (24), the Beck Anxiety Inventory (25), the Geriatric Anxiety Inventory (26), and others. The Depression, Anxiety and Stress Scale-21 (27) could measure both depression and anxiety. The Parkinson's Disease Questionnaire-39 (28) was used to evaluate the quality of life of patients. The Parkinson's Disease Questionnaire-8 is a simplified version and both scales contain 8 dimensions of questions. Insomnia was evaluated mainly by the Insomnia Severity Index (29) while sleep quality by the Pittsburgh Sleep Quality Index Score (30). Apathy was measured by the apathy evaluation scale (31) and fatigue was by Fatigue Severity Scale (32). The Inference Questionnaire score (33) was used to assess negative thoughts and the Brief Psychiatric rating scale (34) was to psychiatric rating. Functioning and well-being was evaluated by related dimensions of the Parkinson's Disease Questionnaire-8. Self-efficacy was evaluated with the Stanford Self-Efficacy for Managing Chronic Disease (35). The higher the score was, the more severe the illness was as a result of all scales above except the Parkinson's Disease Questionnaire. Thus, we inverted the outcome of the scale, which measures QOL, to achieve consistency in the analysis so that high scores designated an improvement. These scales above were widely used in clinical practice and have been proved to have sufficient reliability and validity. All the scales used in included studies were listed in the Table 1.

**Table 1:** Main characteristics of studies.

**References**	**Country**	**Study design**	**Mean age**	***N*** **(female %)**	**Duration of PD (Y)**	**Evaluation indicators**	**Intervention**	**CBT completion (%)**	**Frequency of CBT**	**Duration**	**Outcome**
Wuthrich et al. (36)	Australia	RCT (a single center study)	Total = 68.8 ± 9.4	I = 6; C = 5 (36.4%)	Not mentioned	Depression, anxiety, QOL	I = telephone-delivered CBT C = waitlist	100.0	A manualized session once per week	10 w	GDS, GAI, WHO QoF
Berardelli et al. (37)	Italy	RCT	I = 60.5 ± 5.6 C = 57.1 ± 5.3	I = 9; C = 9 (38.9%)	I = 7.1 ± 32.4 C = 5.2 ± 2.0	Depression, anxiety, apathy, QOL	I = group CBT C = psychoeducational	90.0	A 90-min session once per week	12 w	HAM-D, HAM-A, TAEC, PDQ8
Patel et al. (38)	USA	RCT (a single center study)	I = 63.1 ± 6.8 C = 64.5 ± 9.5	I = 14; C = 14 (42.9%)	Not mentioned	Insomnia, fatigue, daytime sleep, depression, QOL	I = computerized CBT C = standard sleep hygiene education	57.1	A sleep log once per day	6 w	ISI, PIRS20, ESS, PHQ-9, FSS, PDQ8
Kuhlman et al. (39)	Switzerland	RCT	I = 65.0 ± 8.7 C = 67.0 ± 11.0	I = 16; C = 14 (23.3%)	I = 15.0 ± 8.9 C = 18.0 ± 8.6	QOL	I = group CBT C = health enhancement program (HEP)	100.0	A 2-h treatment session once per week	9 w	BELA, FKK, PDQ-39
Calleo et al. (40)	USA	RCT	Total = 62.9 ± 7.3	I = 10; C = 6 (12.5%)	Not mentioned	Depression, anxiety	I = CBT C = enhanced usual care (EUC)	70.0	8 skill-based sessions across 12 weeks	12 w	SIGH-D, SIGH-A
Troeung et al. (41)	Australia	RCT	I = 68.0 ± 7.7 C = 62.0 ± 8.3	I = 11; C = 7 (33.3%)	I = 5.7 ± 5.50 C = 4.3 ± 3.2	Depression, anxiety, QOL	I = CBT C = waitlist	90.9	A 2-h session once per week	8 w	DASS, CCL-D, CCL-A, PDQ-39
Rios Romenets et al. (42)	Canada	RCT (a three-arm study)	I = 64.5 ± 16.3 C = 69.5 ± 10.5	I = 6; C = 6; (0.91%)	I = 5.2 ± 1.8 C = 5.2 ± 4.4	Insomnia, sleep quality, fatigue, depression, QOL	I = CBT with bright light therapy C = bright light therapy	100.0	A 90-min session once per week	6 w	SCOPA, ISI, PDSS, PSQI, BDI, KFSS, PDQ-39
Okai et al. (43)	London	RCT	I = 59.3 ± 8.1 C = 57.9 ± 9.5	I = 28; C = 17 (31.1%)	I = 10.5 ± 6.0 C = 8.8 ± 5.6	Anxiety, depression	I = CBT with standard medical care C = standard medical care	58.0	12 sessions across 3 months	3 months	BDI, BAI
Dobkin et al. (13)	USA	RCT	I = 63.7 ± 9.9 C = 65.4 ± 11.2	I = 41; C = 39 (40.0%)	I = 6.5 ± 5.5 C = 6.1 ± 5.6	Depression, sleep quality, anxiety, negative thoughts, QOL	I = CBT with clinical monitoring C = clinical monitoring	88.0	A 60 to 75-min session once per week	10 w	HAM-D, BDI, HAM-A, PSQI, IQ, MSHS
Veazey et al. (44)	USA	RCT	I = 66.0 ± 9.9 C = 75.0 ± 6.1	I = 5; C = 5 (0%)	Not mentioned	Depression, anxiety, QOL	I = telephone-administered CBT C = support strategy	80.0	A session once per week	9 w	PHQ-9, BAI, PDQ-39
Rodgers et al. (45)	Australia	RCT	Total = 63.7 ± 8.8	I = 18; C = 18 (44.4%)	Not mentioned	Depression, anxiety, QOL	I = MBCT C = waitlist	88.2	6 2-h sessions across 8 weeks	8 w	GDS, DASS-D, GA, DASS-A, PDQ-39
Ghielen et al. (46)	Netherlands	RCT	I = 59.6 ± 9.7 C = 66.6 ± 8.4	I = 19; C = 19 (39.1%)	I = 10.5 ± 5.7 C = 12.3 ± 4.3	Depression, anxiety, QOL	I = body awareness intervention C = treatment as usual	87.0	A 1-h session twice per week	6 w	BDI, BAI, PDQ-39
Kraepelien et al. (47)	Sweden	RCT	I = 65.9 ± 8.5 C = 66.1 ± 9.8	I = 38; C = 39 (39.0%)	I = 8.3 ± 4.4 C = 9.6 ± 5.7	Depression, anxiety, insomnia, function and well-being, self-efficacy, QOL	I = internet-based CBT C = waitlist	91.9	A module once per week	10 w	HADS-D, HADS-A, BBQ, ISI, PDQ-8, SSES6
Dobkin et al. (48)	USA	RCT	I = 65.6 ± 9.8 C = 64.8 ± 9.6	I = 37; C = 35 (48.6%)	I = 7.0 ± 7.8 C = 5.7 ± 4.2	Depression, anxiety, QOL	I = CBT with treatment as usual C = treatment as usual	89.2	A session once per week	3 months	HAM-D, BDI, HAM-A, MOSSF,

*BAI, Beck Anxiety Inventory; BDI, Beck Depression Inventory; BELA, the Burden Questionnaire for Patients with Parkinson's Disease; BBQ, the Brunnsviken Brief Quality of life scale; CCL-A, Cognitions Checklist-Anxiety; CCL-D, Cognitions Checklist-Depression; CBT, cognitive behavioral therapy; C, control group; DASS, Depression, Anxiety and Stress Scale; ESS, Epworth Sleepiness Scale; FKK, The Questionnaire for Disease-Related Communication; FSS, Fatigue Severity Scale; GAI, Geriatric Anxiety Inventory; GDS, Geriatric Depression Scale; HAM-A, Hamilton Rating Scale for Anxiety; HAM-D, Hamilton Rating Scale for Depression; HADS-D, the Hospital Anxiety and Depression Scale; HADS-A, the Hospital Anxiety and Depression Scale; IQS, Inference Questionnaire Score; ISI, Insomnia Severity Index; I, intervention group; KFSS, Krupp Fatigue Severity Scale; MBCT, mindfulness-based cognitive therapy; MOSSF, Medical Outcomes Study Short Form-36; N, number of the participants; PDQ-39, The Parkinson's Disease Questionnaire-39; PDQ-8, the Parkinson's Disease Questionnaire-8; PDSS, Parkinson's Disease Sleep Scale; PHQ-9, Patient Health Questionnaire; PIRS20, Pittsburgh Insomnia Rating Scale; PSQI, Pittsburgh Sleep Quality Index; QOL, quality of life; RCT, randomized controlled trial; SCOPA, Scales for Outcomes in Parkinson's Disease-Night; SIGH-A, Structured Interview Guide for the Hamilton Anxiety Scale; SIGH-D, Structured Interview Guide for the Hamilton Depression Scale; SSES6, the Stanford Self-Efficacy for Managing Chronic Disease; TAEC, The Apathy Evaluation Scale; WHO QoF, World Health Organization Quality of Life-Brief; w, week; Y, year*.

### Assessment of Quality of Literature

Two independent authors used the Cochrane Collaboration risk-of-bias assessment tool to detect the bias in the included papers, including random sequence generation, allocation concealment, blinding of participants and personal, blinding of outcome assessment, incomplete outcome data, selective reporting, and other bias falling under the seven domains (49). Each bias is divided into three level containing low risk, unclear risk, and high risk of bias. We gained the total quality assessment by assessing each of the included study after reading it.

### Data Analysis

The main analysis compared the differences between the two groups post-intervention to estimate the efficacy of CBT. Required data for analysis included means and standard deviations (SD). The inclusion studies provided the scores before and after the intervention, and we analyzed the change scores calculated according to the formula [Computational Formula in the Appendix (Supplementary Material)] (50). This study used the random-effects model. We obtained the pooled efficacy in standard mean differences (SMDs) and 95% confidence intervals using the various scales in the included studies, considering 0.2 as a small effect size, 0.5 as a moderate effect size and 0.8 as a large effect size. The *P*-value was used to determine whether the result was meaningful. When *p* < 0.5, the results were considered to be meaningful. The I-squared and *p*-value in the forest plot were a standard for the heterogeneity.

We reported comparability across the studies using *I*^2^ statistics to evaluate study heterogeneity. When *I*^2^ <30%, we consider it to have mild heterogeneity, when *I*^2^ <60%, it is moderately heterogeneous, and when *I*^2^ >60%, it is highly heterogeneous. We performed a subgroup analysis of the data according to the different subgroups to compare which type of intervention would have a better effect. And sensitivity analysis and subgroup analysis as well were used to find and reduce heterogeneity. Publication bias was evaluated by funnel plot and Egger's regression intercept. We used State 16.0 for the graphs.

## Results

### Literature Search

We found 1,551 related articles through on-line databases, among which 469 repetitive articles were then excluded. When the word PD was retrieved, studies would be presented as long as the word appeared in their title and abstract. Therefore, some articles would be included for this reason and needed to be screened out again with “not PD.” Same thing with CBT that should be screened out again with “non-CBT.” Then we excluded 985 non-conforming articles. After reading through the titles and abstracts, we deleted 69 articles that did not meet the eligibility criteria.

**Figure 1:**
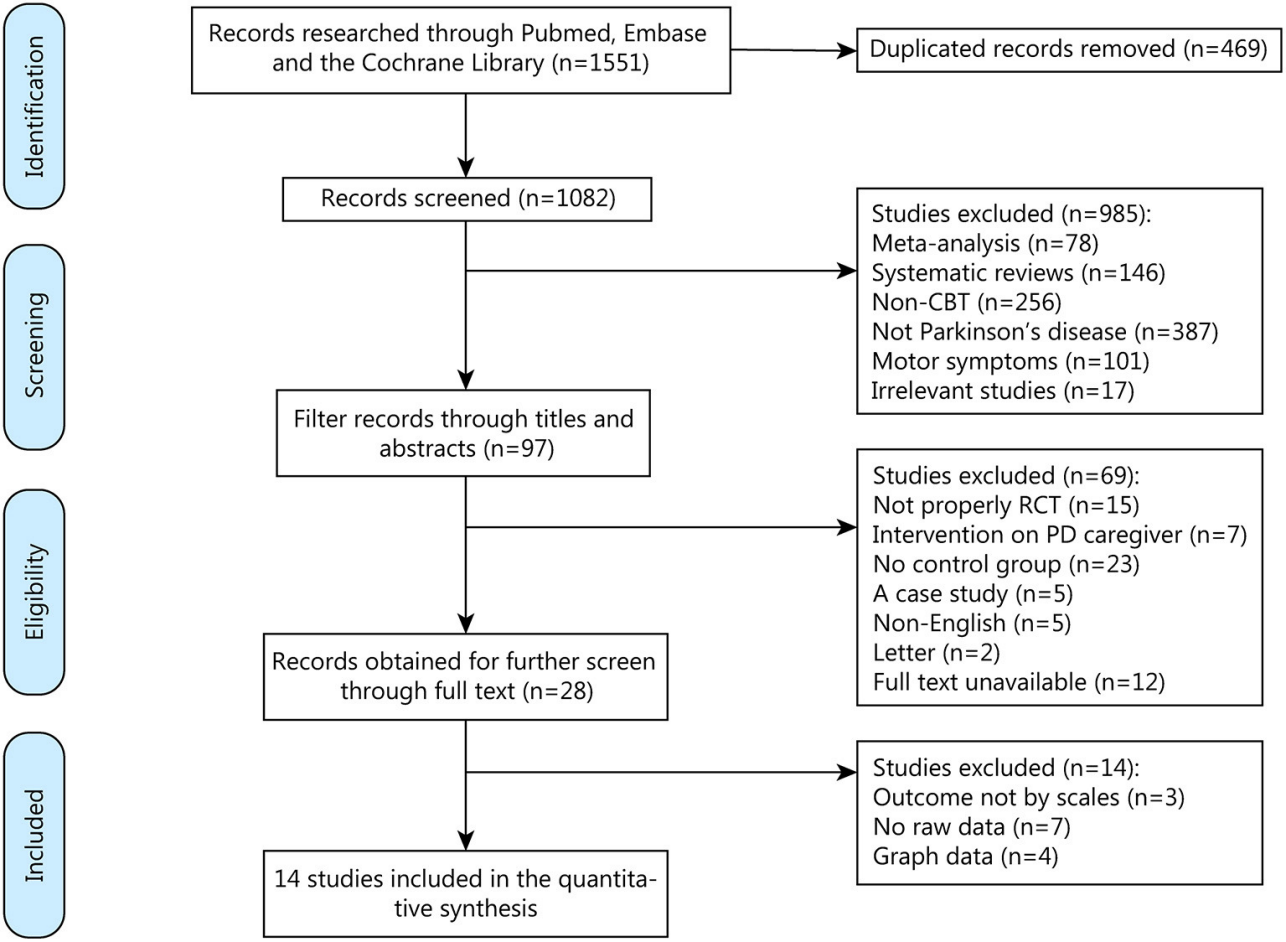
Screening process for trials included in the meta-analysis. CBT, cognitive behavioral therapy; RCT, randomized controlled trial; PD, Parkinson's Disease.

We acquired the full text of 28 articles and eliminated 14 of them for the specific reasons described of which three for outcome not by scales, seven for no raw data while 4 for graph data (Figure 1). Finally, 14 qualifying articles published between 2009 and 2021, comprising 507 participants, were analyzed in this meta-analysis.

### Characteristics of Publications

The basic characteristics of the publications are shown in Table 1. Among the qualified articles, in 12 studies the efficacy of CBT was evaluated, while one study performed Mindfulness-based Cognitive Therapy (MBCT) and one performed Acceptance and Commitment Therapy (ACT). The sample participants from Europe, Australia and North America were 63.3 years old, on average, and comprised 36.3% women. Patients' non-motor symptoms covered depression, anxiety, sleep disorders (insomnia, sleep quality, and daytime sleep), fatigue and others. Five articles failed to mention the duration of PD, and only 4 studies reported the Hoehn & Yahr status of patients (one reported a staging of >I, two reported II and one reported II-III). The CBT completion rate in most studies was above 80%. Only three studies had a completion rate below 80% which showed high feasibility. The frequency of CBT and outcome measure scales are displayed in Table 1.

### Risk of Bias

**Figure 2:**
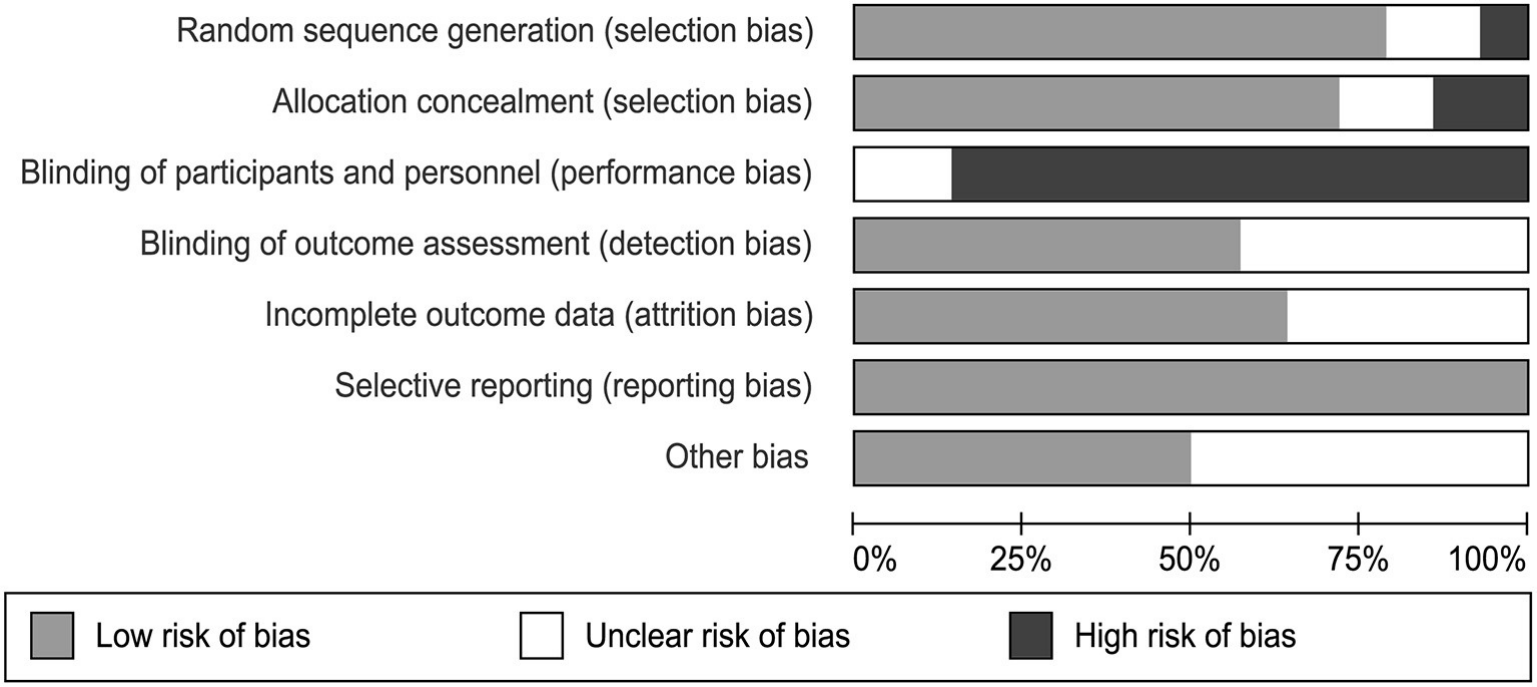
Risk of bias in the included trials.

We evaluated the quality of each study using the Cochrane Collaboration risk-of-bias assessment tool. This tool assessed the risk of a study from six perspectives and marked high, low, or unclear risk (Figure 2). It was objectively difficult to participants to be blind as they have to take CBT session as their treatment. Therefore, we consider studies having a high bias quality when evaluating performance bias. Two studies did not describe the method of random assignment. Six of the studies did not report whether the outcome data was assessed with blindness. There were 5 studies that did not provide process strategies for missing data. All of these are considered unclear risks. Each assessment was conducted by two independent reviewers (FL and TL).

### Synthesis of Results

**Figure 3:**
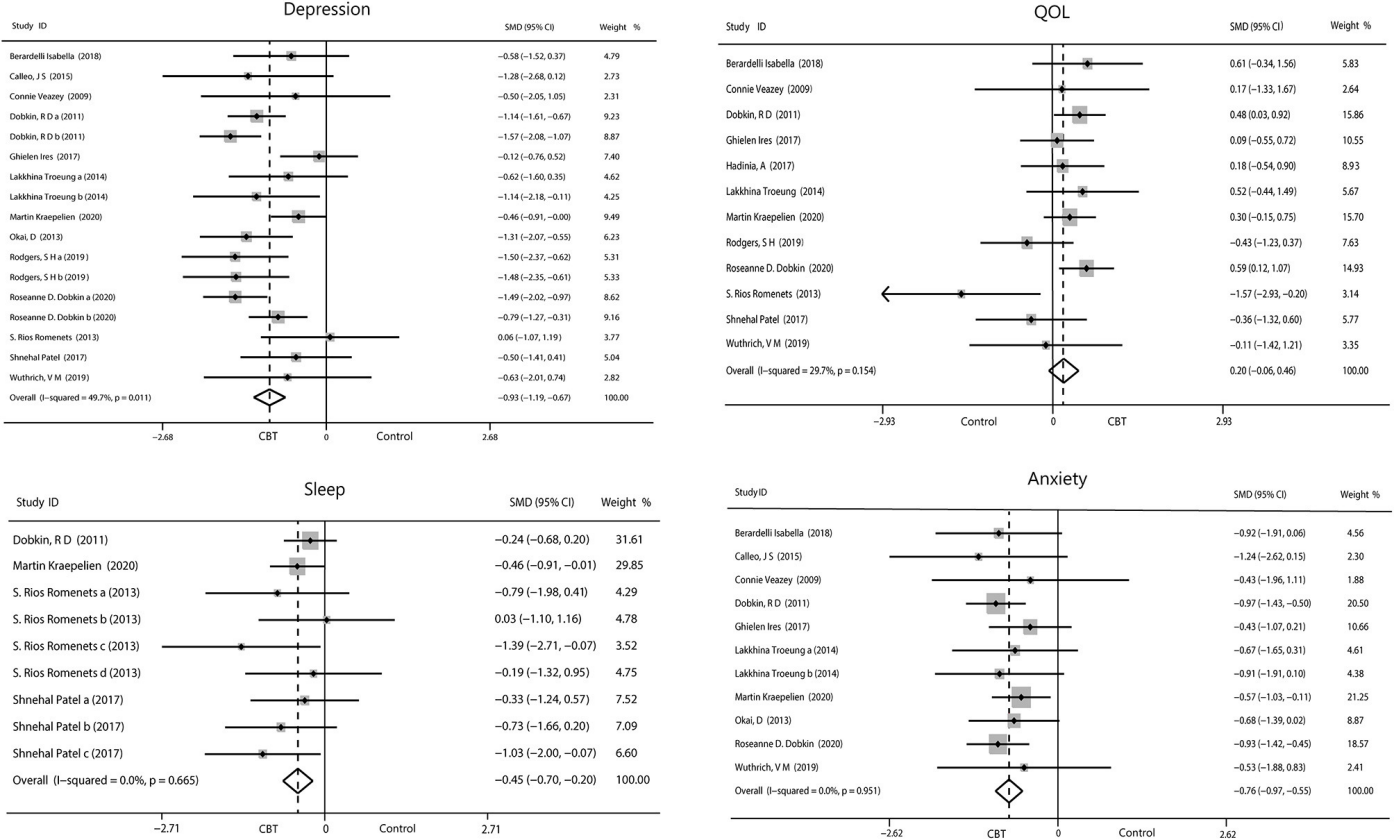
Forest plot of random effects model meta-analysis of the effect of CBT on depression, anxiety, sleep, and QOL. CBT, cognitive behavioral therapy; QOL, quality of life.

**Figure 4:**
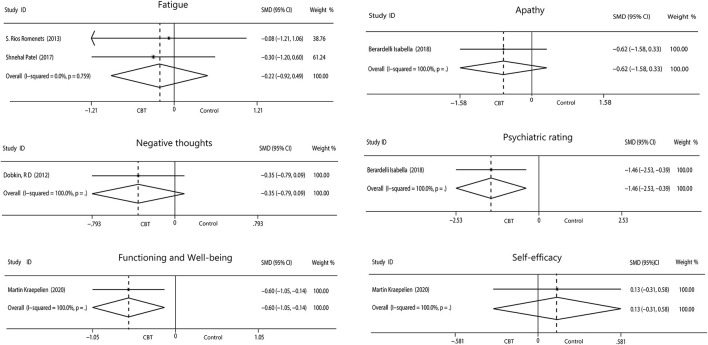
Forest plot of random effects model meta-analysis of the effect of CBT on fatigue, apathy, negative thoughts, psychiatry rating, functioning, and well-being and self-efficacy. CBT, cognitive behavioral therapy.

We evaluated the non-motor symptoms with the provided data at post-treatment. Patients in the 13 studies reporting depression displayed large improvement in depression (−0.93, 95%CI −1.19 to −0.67, *P* < 0.001, *I*^2^ = 49.7%) after intervention (Figure 3). Significant effects of CBT were also found in anxiety (−0.76, 95%CI −0.97 to −0.55, *P* < 0.001, *I*^2^ = 0%; Figure 3) as reported by 10 studies. There were four studies related to sleep disorders in which CBT was noted to have had a moderate effect (−0.45, 95%CI −0.70 to −0.20, *P* = 0.0004, *I*^2^ = 0%) upon completion of the study (Figure 3). There was quite slight impact of CBT on fatigue (−0.22, 95%CI −0.92 to 0.49, *P* = 0.55, *I*^2^ = 0%; Figure 4) and quality of life (0.20, 95%CI −0.06 to 0.46, *P* = 0.12, *I* ^2^= 29.7%; Figure 3). There was a set of data about apathy (−2.50 vs. 1.60), negative thoughts (−1.95 vs. −0.73), psychiatry rating (−6.20 vs. 0.3), functioning and well-being (−0.54 vs. 1.67) showing that the intervention group had more improvement than the control group while the self-efficacy (0.2 vs. −1.25) did not (Figure 4). Within all of the symptoms the heterogeneity was 0%, except for depression and QOL, with a heterogeneity of 49.7 and 29.7%, respectively. One study was removed from the assessment of anxiety because it increased the *I*^2^*-*value to 69%. Our rationale for this exclusion is described below. We have put all the results in the Table 2.

**Table 2:** Pooled effects of CBT on the mood disorders, sleep, fatigue, and QOL in PD patients.

		***N***	**SMD [95% CI]**	***I*** **^2^**	***p***			***N***	**SMD [95% CI]**	***I*** **^2^**	***p***
**Depression**											
All studies		621	−0.93 [−1.19, −0.67]	50	<0.001						
Duration	<8 w	70	−0.19 [−0.66, 0.28]	0	0.43	Region	Europe	168	−0.57 [−1.04, −0.11]	48	0.02
	8–10 w	97	−1.16 [−1.61, −0.70]	0	<0.001		North America	354	−1.05 [−1.39, −0.70]	48	<0.001
	>10 w	454	−1.05 [−1.36, −0.74]	52	<0.001		Australia	99	−1.16 [−1.60, −0.72]	0	<0.001
Treatment format	Group	104	−0.41 [−0.81, −0.01]	0	0.04	Delivery methods	Off-line	193	−0.84 [−1.28, −0.40]	50	<0.001
	Non-group	517	−1.09 [−1.36, −0.82]	43	<0.001		On-line	257	−0.84 [−1.28, −0.40]	47	0.0002
**Anxiety**											
All studies		383	−0.76 [−0.97, −0.55]	0	<0.001						
Duration	<8 w	38	−0.43 [−1.07, 0.21]	–	0.19	Region	Europe	168	−0.60 [−0.91, −0.28]	0	0.0002
	8–10 w	43	−0.72 [−1.36, −0.09]	0	0.03		North America	170	−0.94 [−1.26, −0.62]	0	<0.001
	>10 w	302	−0.81 [−1.05, −0.57]	0	<0.001		Australia	45	−0.73 [−1.35, −0.11]	0	0.02
Treatment format	Group	92	−0.65 [−1.08, −0.23]	0	0.003	Delivery methods	Off-line	127	−0.66 [−1.03, −0.30]	0	0.0004
	Non-group	291	−0.80 [−1.04, −0.55]	0	<0.001		On-line	165	−0.72 [−1.03, −0.40]	0	<0.001
**Sleep**											
All studies		265	−0.45 [−0.70, −0.20]	0	0.0004						
Insomnia		141	−0.66 [−1.01, −0.32]	0	0.0002	Duration	<8 w	108	−0.61 [−1.01, −0.21]	0	0.003
Sleep quality		104	−0.20 [−0.59, 0.18]	0	0.3		>10 w	157	−0.35 [−0.66, −0.03]	0	0.03
Treatment format	Group	48	−0.52 [−1.13, 0.08]	3	0.09	Delivery methods	Off-line	48	−0.52 [−1.13, 0.08]	3	0.09
	Non-group	217	−0.43 [−0.70, −0.16]	0	0.002		On-line	137	−0.55 [−0.90, −0.21]	0	0.002
**QOL**											
All studies		404	0.20 [−0.06, 0.46]	30	0.12						
Duration	<8 w	68	−0.44 [−1.28, 0.40]	57	0.3	Varied scales	PDQ	315	0.14 [−0.16, 0.45]	37	0.36
	8–10 w	80	0.06 [−0.39, 0.51]	0	0.79		Others	89	0.42 [−0.01, 0.84]	0	0.05
	>10 w	256	0.44 [0.20, 0.69]	0	0.0005						
**Fatigue**						**Apathy**					
All studies		32	−0.22 [−0.92, 0.49]	0	0.55	All studies		18	−0.62 [−1.58, 0.33]	–	0.2
**Negative thoughts**						**Psychiatric rating**					
All studies		82	−0.35 [−0.79, 0.09]	–	0.12	All studies		18	−1.46 [−2.53, −0.39]	–	0.008
**Functioning and well-being**						**Self-efficacy**					
All studies		77	−0.60 [−1.05, −0.14]	–	0.01	All studies		77	−0.13 [−0.31, 0.58]	–	0.56

*CBT, cognitive behavioral therapy; N, number of participants; PDQ, The Parkinsons Disease Questionnaire; QOL, quality of life; SMD, standard mean difference; w, week*.

### Subgroup Analysis

We performed a subgroup analysis of depression, anxiety, sleep, and quality of life, based on the nationality of participants (America, Europe, and Australia), duration of intervention (<8 weeks, 8–10 weeks, and >10 weeks), treatment format (group and non-group), and delivery methods (on-line including telephone and computer, off-line including in-person). When analyzing delivery methods interventions, we excluded two studies that used both methods. Fatigue was not included as a symptom in the final analysis for lacking of sufficient data.

#### Depression

**Figure 5:**
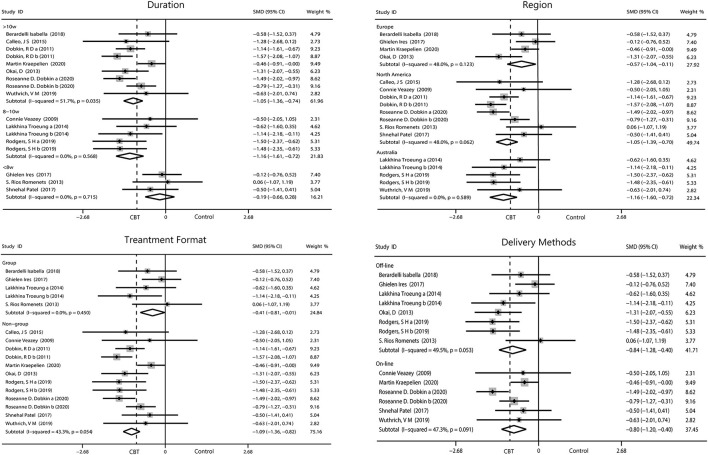
Forest plot of random effects model meta-analysis of the effect of CBT on subgroups of depression. CBT, cognitive behavioral therapy.

Duration of CBT intervention was divided into <8 weeks (−0.19, 95%CI −0.66 to 0.28, *P* = 0.43, *I*^2^ = 0%), 8–10 weeks (−1.16, 95%CI −1.6 to −0.72, *P* < 0.001, *I*^2^ = 0%), and ≥10 weeks (−1.05, 95%CI −1.36 to −0.74, *P* < 0.001, *I* ^2^= 51.7%). Our results showed that more than 8 weeks of intervention was more advantageous. The effects of all three areas were all significant. Intervention on-line (−0.80, 95%CI −2.01 to −0.40, *P* < 0.001, *I*^2^ = 47.3%) had a similar effect with off-line (−0.84, 95%CI −1.28 to −0.40, *P* = 0.0002, *I*^2^ = 49.5%), and both had a moderate heterogeneity. Individual intervention (−1.09, 95%CI −1.36 to −0.82, *P* < 0.001, *I*^2^ = 43.3%) resulted in greater improvement, compared with group intervention (−0.41, 95%CI −0.81 to −0.01, *P* = 0.04, *I*^2^ = 0%; Figure 5).

#### Anxiety

**Figure 6:**
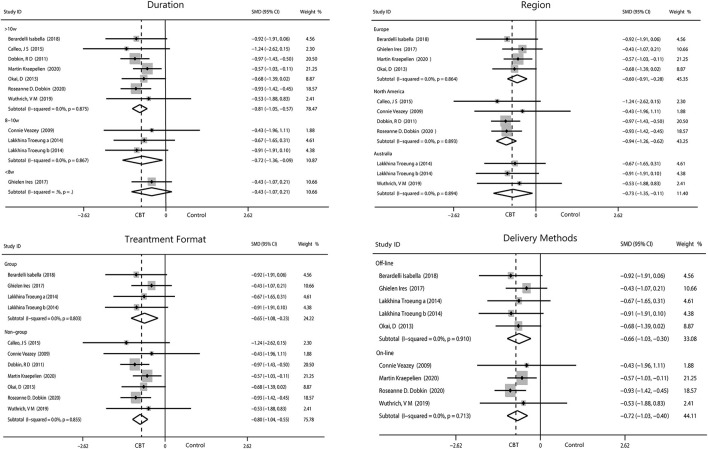
Forest plot of random effects model meta-analysis of the effect of CBT on subgroups of anxiety. CBT, cognitive behavioral therapy.

We performed four subgroup analyses as described. The result showed intervening for more than 10 weeks (−0.81, 95%CI −1.05 to −0.57, *P* < 0.001, *I*^2^ = 0%) obtained the optimum reduction in anxiety. North American patients had the greatest improvement in anxiety (−0.94, 95%CI −1.26 to −0.62, *P* < 0.001, *I*^2^ = 0%) compared with those from Europe and Australia. Intervention off-line and on-line reported equal effect toward the anxiety while intervention in person were more useful than the other method (−0.80, 95%CI −1.04 to −0.55, *P* < 0.001, *I*^2^ = 0%; Figure 6).

#### Sleep

**Figure 7:**
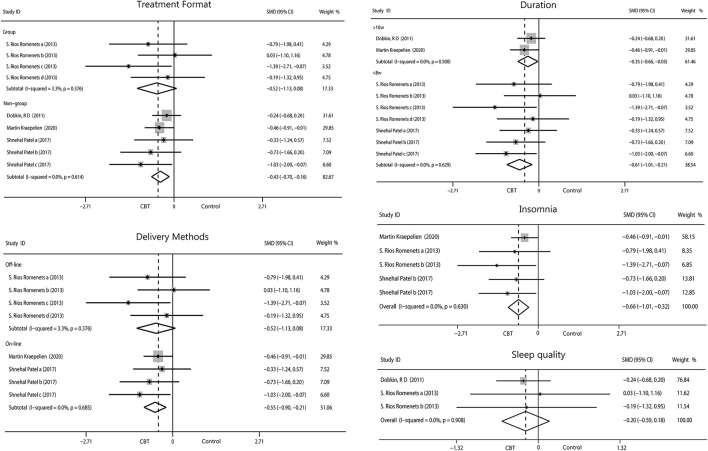
Forest plot of random effects model meta-analysis of the effect of CBT on subgroups of sleep disorders. CBT, cognitive behavioral therapy.

Patients who participated in sleep assessments were all from North America and were only treated for 6 or 10 weeks. We found that the 6-week intervention (−0.61, 95%CI −1.01 to −0.21, *P* = 0.003, *I*^2^ = 0%) showed better outcomes than the 10-week intervention (−0.35, 95%CI −0.66 to −0.03, *P* = 0.03, *I*^2^ = 0%). No significant difference between individual and group intervention or on-line and off-line intervention was found, with all showing medium effect. Sleep covered two items, insomnia (−0.66, 95%CI −1.01 to −0.32, *P* = 0.0002, *I*^2^ = 0%) and sleep quality (−0.20, 95%CI −0.59 to 0.18, *P* = 0.30, *I*^2^ = 0%). We found that CBT had medium improvement on insomnia but only mild increase in sleep quality (Figure 7).

#### Quality of Life

**Figure 8:**
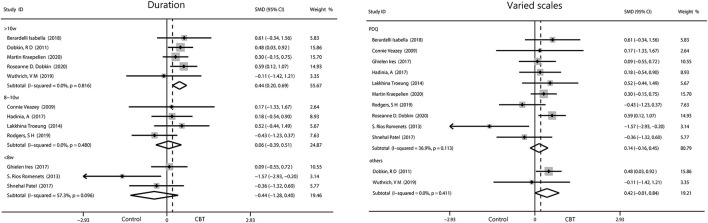
Forest plot of random effects model meta-analysis of the effect of CBT on subgroups of QOL. CBT, cognitive behavioral therapy; QOL, quality of life; PDQ, Parkinson's Disease Questionnaire.

The pooled results on the effect of CBT on QOL in patients with PD had 29.7% heterogeneity, and we tried to discover the source of heterogeneity and mitigate its effect. We obtained a more reliable result (0.30, 95%CI 0.09–0.50, *P* = 0.004, *I*^2^ = 0%) after removing one article (Rios Romenets et al.). Only interventions lasting more than 10 weeks were considered to have a moderate effect on QOL (0.44, 95%CI 0.02–0.69, *P* = 0.0005, *I*^2^ = 0%). No significant results were found by regions and by intervention methods. We analyzed the scales used and found moderate effects were reported using scales other than the PDQ (0.42, 95%CI −0.01 to 0.84, *P* = 0.05, *I*^2^ = 0%), while we found small change score of CBT in enhancing QOL when measured with the PDQ (Figure 8).

### Publication Bias

**Figure 9:**
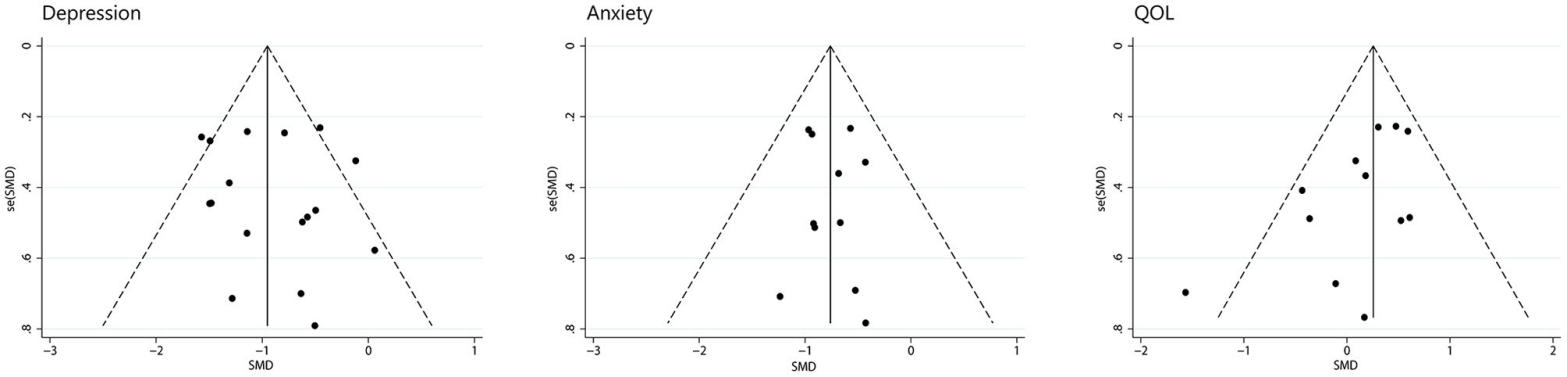
Funnel plot for publication bias evaluation.

We assessed depression, anxiety, and quality of life for publication bias (Figure 9). There was too little data on other symptoms to analyze. As the funnel plot shown, studies of depression and anxiety are evenly distributed on either side of the midline. A egger's regression intercept was performed on the funnel plot as a formal test, which allowed us to determine that there was no evident publication bias for depression (*P* = 0.598) and anxiety (*P* = 0.860). The funnel plot of quality of life was not symmetrical with a corner vacancy on the right, and egger's regression intercept suggests publication bias (*P* = 0.043). Using the Trim and Fill method, the results showed the number of studies unchanged. The effect size after adjustment is the same as before (0.20, 95%CI −0.06 to 0.46), indicating that publication bias has little influence on the results.

## Discussion

Existing data have shown that a variety of non-motor symptoms appear in PD before motor features and even before diagnosis by several years (51, 52). Thus, the symptoms of PD are present throughout the disease course and can have an impact on each of its stages. Symptoms in patients with PD who have higher rates of co-morbid psychiatric and executive dysfunction usually differ from symptoms in patients with non-PD psychiatric disorders (53). Thus, the use of original CBT in patients with PD may have been inappropriate, so CBT protocols have been modified to treat psychological problems in this population. The original CBT mainly includes the following aspects: understanding symptoms and treatment methods, identifying automatic thinking and behavioral activation, combating distorted cognitive and functional behaviors, changing attributional patterns and task decomposition, discovering core beliefs and problem solving, reviewing, and preventing recurrence. Wuthrich and Rapee (36) used *Aging Wisely with Parkinson's Disease* which is a CBT manual that are suitable for participants with PD. The program, while incorporating the core CBT skills, also simplifies the process for cognitive challenging and adds relaxation skills.

In this study, we upgraded prior systematic reviews and meta-analyses by including existing eligible RCT studies, demonstrating that CBT had a considerable efficacy on non-motor symptoms of PD. The quality assessment results of the included literature were fine. This lays a foundation for the reliability of the analysis results.

There is a large body of literature on depression and anxiety due to their high incidence rates in Parkinson's Disease (54, 55). According to the data we collected, CBT greatly reduced the scores for depression (−0.93, 95%CI −1.19 to −0.67) and anxiety (−0.76, 95%CI −0.97 to −0.55). One article by Rodgers et al. with a high level of heterogeneity was excluded from the analysis of anxiety. The authors used two scales (DASS-21 and GAI) to assess changes in anxiety. One of the scales had a positive outcome (GAI), while the other had a negative outcome (DASS-21). There was huge heterogeneity between the two sets of data, which would seriously reduce the credibility of our results if we included. The authors suggested that these inconsistencies may have been related to the low baseline level of anxiety in their patients and the type of intervention implemented. We also performed a subgroup analysis on the duration of CBT intervention, which revealed that >8 weeks of CBT was more effective than <8 weeks. We can assume the longer the intervention, the better the outcome. PD is a persistent disease with chronic symptoms, making rapid treatment response difficult, especially because most patients with PD are older (56). CBT programs generally lasted between 6 and 12 weeks in our included studies, excepting one that was 6 months long. Therefore, long-term intervention RCT trials are needed to further explore the effects of CBT. Bomasang-Layno et al. (57) have reported consistent results, but they only included five relevant RCTs with limited data. Our study confirmed this result further.

When analyses were narrowed to the trials in off-line interventions, effect sizes appeared to be almost equal with the on-line (telephone or computer) intervention. Two studies (by Calleo et al. and Dobkin et al.) that included both on-line and off-line CBT approaches were excluded from the subgroup analysis. It is important to note that the subgroup analysis did not reduce heterogeneity, which may have some implications for the interpretation of the results. Eligible studies of on-line CBT had small sample sizes and one study reported a high drop-out rate, which may have altered our results. Problems with face-to-face CBT include limitations in clinician–patient interaction due to patients' physical disability and the time patients spent traveling to appointments (58). These barriers may have contributed to the development of telemedicine. The accessibility of telemedicine reduces the burden and psychological distress of face-to-face CBT and appears to lower attrition (59, 60). Although it was easier to operate, telemedicine didn't seem to have a problem that the elder were difficulty to adapt. There was not much data from on-line studies which may affect the effectiveness of the final merge results.

We also limited our focus on the trials with group CBT interventions, in which effect size was moderate and slightly inferior to non-group intervention, which had a large effect size. Older individuals suffering from PD with psychological disorders are more likely to experience inferiority, stigma and fear because of increased dysfunction (61). Patients in one group of older adults with PD tended to have unequal degrees of disease, and by mutually comparing themselves may have deepened their own withdrawal and enhanced communication difficulties. Moreover, conducting CBT in a group is not targeted enough compared with individual therapy where individuals may fully focus without the distraction of other participants, which makes the efficacy of group intervention limited. In our study, although the improvement in symptoms seen after group CBT intervention was indelible, the non-group CBT intervention had the most significant effect.

We also analyzed outcomes in different regions based on the nationality of the patients included in the studies. These patients were recruited in Europe, North America and Australia. The effect sizes observed in both depression and anxiety were moderate to large. Compared with the other two, the European effect is relatively poor. The reasons for this fact need to be further explored. More specific research is needed to validate these results in populations across other geographic locations.

Sleep disorders are a high-morbidity-rate complication in PD that have a negative impact on patients (62). Our results showed that CBT had a moderate overall effect on sleep disorders (−0.45, 95%CI −0.70 to −0.20). Our subgroup analysis showed no significant difference in the efficacy of different methods of CBT, including on-line or off-line and group or non-group interventions, on sleep disorders. Because all forms of CBT intervention showed at least a moderate effect size, the intervention forms had no interactions with the results. According to the subgroup analysis of the intervention duration, the efficacy of the intervention for 10 weeks was inferior to that of the intervention for 6 weeks, which is contrary to what we have always thought and is not consistent with the results of previous studies (63). We tried to guess the reason. There were seven sets of data at 6 weeks of intervention, but they came from only two studies. Both studies had small sample sizes, which made the results unreliable. We separately analyzed the change in insomnia post-intervention, which resulted in a medium effect size (−0.66, 95%CI −1.01 to −0.32). It has reported CBT is beneficial to insomnia in patients with or without PD (64). This may be because insomnia has been associated more with persistent psychological factors than specific disease characteristics in the general population (65), while the goal of CBT in treating insomnia is to alter erroneous perceptions, cognitive arousal, and maladaptive behaviors toward sleep hygiene (66). Another result revealed that sleep quality did not ameliorate, which demonstrates that CBT can influence sleep duration rather than sleep architecture. Only one set of data on daytime sleep was provided in one of the included studies, precluding the ability to run a separate analysis. The data showed daytime sleep was reduced after CBT.

Assessments of both sleep and fatigue are often performed simultaneously. We analyzed the effect of CBT on fatigue, which showed there was no evidence of a marked reduction in fatigue after CBT. Physical fatigue caused by dyskinesia that cannot be alleviated by CBT may be one of the reasons for the small improvement, suggesting that motor and non-motor symptoms potentially synergize, and treatment of a single symptom may undermine optimal efficacy. Interestingly, as the actions of the sleep homeostat, short periods of nocturnal sleep and may cause daytime sleep or fatigue, implying a connection exists between sleep and fatigue as symptoms (63). We can infer that improvements can be detected simultaneously in these symptoms after CBT.

Our results showed quality of life did not improve significantly for patients with PD after CBT (0.20, 95%CI −0.06 to 0.46). One of the articles had increased heterogeneity that may have been related to the inclusion of light therapy. Patients were allowed to read about the mechanisms of light therapy independently, and therefore they were not completely blinded to the intervention in this study. Several scales were used to assess the quality of life. The PDQ is a questionnaire with 39 questions in eight dimensions (67). The PDQ contains many questions about physical problems and/or mobility difficulties that cannot be solved through psychological therapy, a possible reason for the lack of improvement in quality of life after CBT. We performed a subgroup analysis of the scales used to measure quality of life and found a moderate effect on quality of life in studies that did not use the PDQ, while there was no significant efficacy of CBT on quality of life in studies that used the PDQ. This may suggest that the choice of scales had an influence on the evaluation results of quality of life. We also found the duration of the intervention affected results, as group CBT interventions for >10 and <8 weeks yielded moderate effect sizes on quality of life. However, outcome of studies which intervene <8 weeks reported heterogeneity of 57.3% that reduce the accuracy of the effect. Previous literature has reported that depression, anxiety, sleep disorder, and apathy are predictors of quality of life (39). However, in our study, although we observed an improvement in depression, anxiety and sleep disorder after CBT, quality of life did not particularly improve. The relationship between non-motor symptoms and quality of life in PD remains to be explored.

In addition, one article dealt with apathy and psychiatry rating, one with negative thoughts, another one with functioning and well-being and self-efficacy, and each of the five indicators had only a set of data. Limited data have shown that the intervention group had a more desired effect and that CBT improved outcomes compared to the control group. On the contrary, self-efficacy outcomes were better in the control group. Any one of these symptoms requires more research data to explore more reliable results.

There were only three studies with a completion rate below 80%. One of the studies reported a lower completion rate because older adults could not get used to using computers, but the other study, which also used computers to complete the intervention, had a completion rate of 91.9%. The exact reasons for this are still to be explored. And at the same time, it is indeed difficult to use computers in the elderly, which is a problem that needs to be solved. Overall, the feasibility of CBT is quite high. Our study did not analyze the mid-term and long-term effects of CBT on non-motor symptoms due to insufficient data. Mid-term effects can provide us with details about how the patient's symptoms change during the intervention which had great implications for long-term intervention. Studies had reported moderate long-term effects of CBT on psychological symptoms such as depression and anxiety (68), but few studies had been done on patients with PD. Parkinson's non-motor symptoms tend to persist for a long time, and as chronic symptoms, it may be difficult to respond quickly to the treatment. Thus, we speculate that there is a long-term benefit of CBT for the non-motor of Parkinson's disease. However, long-term follow-up is often limited by many difficulties, such as increased rate of drop-out, deterioration of underlying diseases and some unexpected accidents. Therefore, adequate preparation is necessary before research.

We obtained a high bias when assessing performance bias. As CBT is designed to improve symptoms through courses and training which patients can learn about from their manuals, they can not be completely blind. This may have resulted in the treatment effects of the intervention group being exaggerated which reflected on the self-rating scale, thus skewing the results in favor of CBT group. This is actually hard to avoid. No publication bias was found for depression and anxiety after assessment. Quality of life showed a certain publication bias, but it was proved to have little effect on the results after using the Trim and Fill method which suggested that result is stable.

All of the studies in this analysis were RCTs. The missing data is obtained by contacting the author of the original text. The studies reported no serious adverse events. Our quantitative analysis revealed little heterogeneity among studies and after exploring possible reasons, the two papers causing the increased heterogeneity were removed from analysis. The scales used to measure the outcomes of the included studies are accredited and have been used extensively in clinical practice, such as the Hamilton Rating Scale (HAM), Beck Anxiety Inventory (BAI) and others. We chose the random effects model to eliminate the discrepancy between scales to ensure reliable results.

## Limitations

This analysis had several limitations. First, the sample sizes of the included studies were generally small, with the largest sample having only 80 participants, which may lead to a lack of generalizable results. We need wider clinical trials to perform further research. Second, inadequate data on apathy, negative thoughts and psychiatric rating prevented us from making a comprehensive assessment of these indexes. More relevant studies need to be included to obtain more reliable quantitative results. It is necessary to exploit more convenient and suitable CBT intervention methods for patients with PD. The effect of long-term CBT intervention on non-motor symptoms and quality of life needs further study. Thirdly, it is vital to notice that there was a certain heterogeneity in the control group interventions in each study. Some participants are in the waiting-list while others are provided with standard medical treatment or placebo. Although they found no difference in effect size before and after treatment, the lack of complete consistency in control conditions lead to the limitation of the accuracy of comparisons. Finally, the measurement scales used in the selected studies were validated but highly varied, resulting in the possibility that an outcome might be exaggerated or minimized during the process of data merging (17). Gathering symptom data using the same scales across studies would result in better compatibility and potentially more accurate results.

## Conclusion

Cognitive behavioral therapy had a significant effect on depression, anxiety, sleep disorders and some other non-motor symptoms in patients with PD, while it had no effect on fatigue or quality of life. The longer the intervention, the greater the effect. Symptom improvements after individual are much more remarkable than after group interventions while no significant difference was observed between on-line and off-line interventions. The use of CBT should be considered as a standard clinical treatment for non-motor symptoms of PD, and more convenient and efficient forms should be explored.

## Data Availability Statement

The original contributions presented in the study are included in the article/Supplementary Material, further inquiries can be directed to the corresponding author/s.

## Author Contributions

FL designed the study, managed the literature searches, analyzed and review statistical data, and wrote the draft of the manuscript. MY and CQ designed the study, review, and critique the statistical data and manuscript. TL managed the literature searches, analyzed, and reviewed statistical data. ZL designed the study and review and critique the manuscript. BH, JC, JY, AW, FC, and ZH managed the literature searches and analyses. ZD, JZ, and CQ modified the manuscript. All authors contributed to and have approved the final manuscript.

## Funding

This work was funded by Zhejiang Medical Health Science and Technology Project (Nos. 2019KY724 and 2020KY332), the Scientific Research Fund of Shaoxing University (No. 20125025), and the National Training Program of Innovation and Entrepreneurship for College Students (No. 2017R10349001).

## Conflict of Interest

The authors declare that the research was conducted in the absence of any commercial or financial relationships that could be construed as a potential conflict of interest.

## Publisher's Note

All claims expressed in this article are solely those of the authors and do not necessarily represent those of their affiliated organizations, or those of the publisher, the editors and the reviewers. Any product that may be evaluated in this article, or claim that may be made by its manufacturer, is not guaranteed or endorsed by the publisher.
