# Supplementary material for: Efficacy of Cognitive Behavioral Therapy on Mood Disorders, Sleep, Fatigue, and Quality of Life in Parkinson's Disease: A Systematic Review and Meta-Analysis
Source: Front Psychiatry. 2021 Dec 13;12:793804. doi: 10.3389/fpsyt.2021.793804 (PMC8710613; doi:10.3389/fpsyt.2021.793804)
Supplement: Supplementary file 1 [file Data_Sheet_1.DOC]

Supplementary Appendix

Search Strategy..............................................................................................................2

Computational Formula.................................................................................................3

**Search Strategy**

1. Parkinson's Disease OR Parkinson's Disease, Idiopathic OR Parkinson's Disease, Lewy Body OR Idiopathic Parkinson's Disease OR Lewy Body Parkinson's Disease OR Primary Parkinsonism OR Parkinsonism, Primary OR PD
2. Cognitive Behavioral Therapy OR Behavioral Therapy, Cognitive OR Therapy, Cognitive Behavioral OR Cognitive Behavior Therapy OR Behavior Therapy, Cognitive OR Therapy, Cognitive Behavior OR Cognitive Behavioral Therapies OR Behavioral Therapies, Cognitive OR Therapies, Cognitive Behavioral OR Cognitive Behavior Therapies OR Behavior Therapies, Cognitive OR Therapies, Cognitive Behavior OR Cognition Therapy OR Therapy, Cognition OR Cognition Therapies OR Therapies, Cognition OR Cognitive Therapy OR Therapy, Cognitive OR Cognitive Therapies OR Therapies, Cognitive OR CBT
3. 1 AND 2
4. Mindfulness-based Cognitive Therapy OR MBCT
5. 1AND 4
6. Acceptance and Commitment Therapy OR ACT
7. 1AND 6
8. Dialectical Behavior Therapy OR Behavior Therapy, Dialectical OR Dialectical Behavior Therapies OR DBT
9. 1 AND 8
10. mindfulness-based stress reduction therapy OR MBSR
11. 1 AND 10

**Computational Formula**

Sc^2^=S_B_^2^+S_F_^2^-2R×S_B_×S_F_

S_F_^2^-(-2×R×S_B_)×S_F_+(S_B_^2^-Sc^2^)=0

S_F_={2R×S_B_±√[4R^2^×S_B_^2^-4×(S_B_^2^-Sc^2^)]}/2

R value for 0.5

S_F_=[S_B_±√(4×Sc^2^-3×S_B_^2^)]/2

B:Baseline of standard mean difference

C:Change in standard mean difference

F:Final value of standard mean difference
